# Endovascular repair of diseases originating in the ascending thoracic aorta: A systematic review and meta-analysis

**DOI:** 10.1016/j.xjse.2026.100100

**Published:** 2026-01-24

**Authors:** Jasper F. de Kort, Michiel Been, Nesar A. Hasami, Viviana Grassi, Gabriele Piffaretti, Guido Gelpi, Jorg L. de Bruin, Constantijn E.V.B. Hazenberg, Christopher P. Twine, Robin Heijmen, Anders Wanhainen, Joost A. van Herwaarden, Santi Trimarchi

**Affiliations:** a Cardio Thoracic Vascular Department, Section of Vascular Surgery, Fondazione IRCCS Ca’ Granda Ospedale Maggiore Policlinico, Milan, Italy; b Department of Vascular Surgery, University Medical Centre Utrecht, Utrecht, the Netherlands; c Department of Vascular Surgery, Erasmus Medical Centre, Rotterdam, the Netherlands; d Department of Cardiothoracic Surgery, Radboud University Medical Centre, Nijmegen, the Netherlands; e Department of Medicine and Surgery, Vascular Surgery, University of Insubria Faculty of Medicine and Surgery, Varese, Italy; f Cardio Thoracic Vascular Department, Section of Cardiac Surgery, Fondazione IRCCS Ca’ Granda Ospedale Maggiore Policlinico, Milan, Italy; g Bristol Centre for Surgical Research, SouthMead Hospital, North Bristol NHS Trust, University of Bristol Medical School, Bristol, United Kingdom; h Department of Surgical Sciences, Vascular Surgery, Uppsala University, Uppsala, Sweden; i Department of Clinical Sciences and Community Health, University of Milan, Milan, Italy

**Keywords:** ascending aorta, thoracic endovascular aortic repair, ascending disease, aTEVAR, systematic review

## Abstract

**Objective:**

This report provides a concise overview of the published literature and clinical outcomes on ascending thoracic endovascular aortic repair (aTEVAR) for diseases originating in, but not restricted to, the ascending aorta.

**Methods:**

PubMed, Scopus, and Web of Science were systematically searched for aTEVAR procedures for diseases originating in the ascending aorta. Nonadult patients or studies with fewer than 5 patients were excluded. This review was registered in the International Prospective Register of Systematic Reviews and followed the Preferred Reporting Items for Systematic Reviews and Meta-Analyses statements. The Risk of Bias in Non-randomized Studies - of Interventions tool was used to assess quality. A Grading of Recommendations, Assessment, Development, and Evaluation evidence certainty analysis was performed.

**Results:**

One prospective and 22 retrospective studies included a total of 356 patients (weighted mean age 68.6 ± 10.9 years; 59.3% male). The weighted mean follow-up was 21.2 ± 20.3 months, and prevalent comorbidities were hypertension (72.8%) and coronary artery disease (24.2%). Indications included type A dissections (acute 36.0%; chronic 19.9%), penetrating aortic ulcer (11.5%), and pseudoaneurysm (16.9%). Rapid ventricular pacing (33.4%) was most frequently adopted strategy to reduce cardiac output, and femoral/iliac artery access was used in 84.3%. Tubular stent grafts were used in 229 cases (64.3%), whereas physician-modified stent grafts were necessary in 110 cases (30.9%). In-hospital and follow-up mortality rates were 10.7% (95% CI, 6.3-13.1) and 20.5% (95% CI, 16.4-25.1). Subgroup analyses showed in-hospital and follow-up mortality rates for (acute and chronic) type A dissections of 9.8% (95% CI, 5.9-15.0) and 18.0% (95% CI, 12.7-24.3) subsequently; for penetrating aortic ulcer 9.0% (95% CI, 1.9-24.3) and 15.2% (95% CI, 5.1-31.9); and for pseudoaneurysm 2.8% (95% CI, 1.0-13.9) and 13.3% (95% CI, 5.9-24.6). Intra- and postoperative complications were reported in 43 and 192 cases, with endoleaks occurring in 14 (3.9%) and 37 (10.4%) cases, respectively.

**Conclusions:**

This systematic review assessed current strategies and outcomes for aTEVAR. The findings suggest that aTEVAR holds promise as a treatment option and highlight the potential for further integration of aTEVAR into clinical practice.

**Figure:**
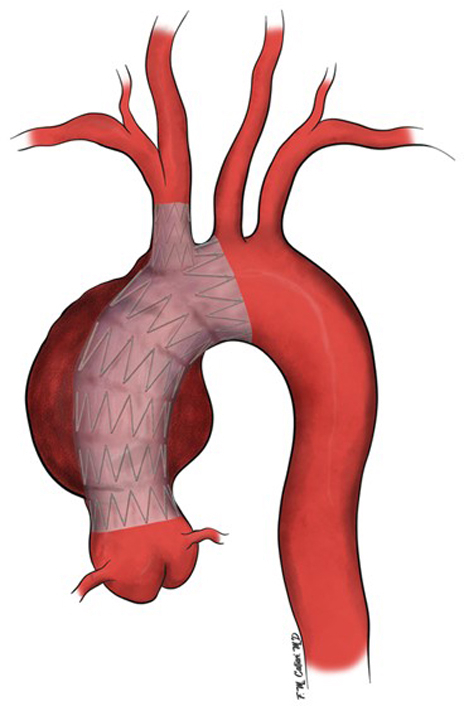
Illustration of endovascular repair of the ascending aorta, extending into the arch.

**Central Message**

aTEVAR is a feasible option for high-risk patients with ascending aortic disease, showing acceptable early mortality but notable complication rates. Dedicated devices and stronger evidence are needed.

**Perspective**

Endovascular repair of the ascending aorta represents a promising option for patients unsuitable for open surgery. Early results show encouraging survival in this high-risk population, but complication rates remain significant. Future progress depends on dedicated device development, procedural standardization, and prospective studies to validate safety and durability.

Thoracic endovascular aortic repair (TEVAR) is the preferred treatment for diseases originating in the distal aortic arch and descending aorta.^1^, ^2^, ^3^, ^4^, ^5^, ^6^ Its minimally invasive nature is associated with lower perioperative and long-term mortality compared with traditional open surgery.^3^, ^4^, ^5^, ^6^, ^7^, ^8^, ^9^ Endovascular repair was first widely adopted for abdominal aortic aneurysms before being extended to the thoracic aorta, initially in the descending segment and later in the arch. In contrast, open surgery remains the gold standard for diseases originating in the ascending aorta.^1^^,^^2^ However, it requires extracorporeal circulation and carries significant morbidity and mortality risks, particularly in elderly patients and in patients who are frail with multiple comorbidities. As a result, many patients are unsuitable for open repair and are instead managed medically. In case of a type A aortic dissection (TAAD), 8% to 28% of patients are reported to be unfit for open repair, with medical treatment resulting in a 30-day mortality of 50% to 81.2%.^10^, ^11^, ^12^, ^13^, ^14^ In such cases, off-label deployment of stent grafts has been increasingly proposed and adopted, as a last resort option, Figure 1.^15^

**Figure 1:**
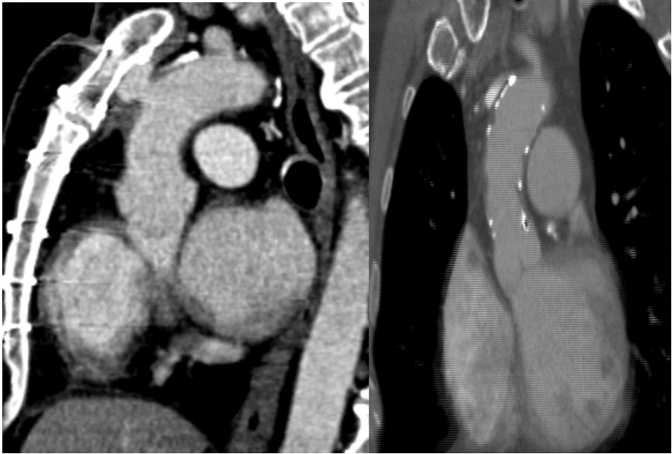
A patient with an aTEVAR without adjunctive procedures for a pseudoaneurysm. The patient had previously undergone cardiac surgery, as indicated by the presence of sternal wires, and presented with a pseudoaneurysm adherent to the manubrium, making open surgery high-risk and aTEVAR a less-invasive treatment option. *aTEVAR*, Ascending thoracic endovascular aortic repair.

The unique anatomical and hemodynamic properties of the ascending aorta pose significant challenges for endovascular repair. These challenges include its short length; proximity to critical structures, such as the coronary ostia, aortic valve, and brachiocephalic trunk; and considerable anatomical variability in curvature and angulation. In addition, its dynamic nature during the cardiac cycle, in terms of radial distensibility and longitudinal extensibility, complicates correct stent graft sizing and deployment.^16^^,^^17^ These challenges are further compounded by the lack of a commercially available stent graft specifically designed for ascending thoracic endovascular aortic repair (aTEVAR) and long-term follow-up data. Previous research by our group focused on aTEVAR procedures confined strictly to the ascending aorta, with stent grafts not extending beyond the origin of the brachiocephalic trunk.^15^ In contrast, the aim of this systematic review is to provide a broader analysis of patient characteristics, procedural specifics, and clinical outcomes of endovascular repair for diseases originating in, but not necessarily limited to, the ascending aorta, thus, emphasizing disease origin rather than stent graft location.

## Methods

### Study Design

The study was registered in the International Prospective Register of Systematic Reviews upon the start of the research (CRD42024544248). Institutional review board or ethics review board approval was not required for this study, because it is a systematic review and meta-analysis of previously published, anonymized data. Written informed consent from patients was therefore not required. The Preferred Reporting Items for Systematic Reviews and Meta-Analyses statements were followed to ensure methodological rigor and transparency.^18^ The quality of evidence and strength of recommendations were evaluated using the GRADE (Grading of Recommendations, Assessment, Development, and Evaluations) system.^19^

### PICO Framework

In accordance with the GRADE approach, the following PICO framework was established before the search strategy: P (patient): diseases in the ascending aorta. I (intervention): endovascular treatment. C (comparison): no comparison group applicable for this research. O (outcome): mortality and complications.

### Information Sources and Search Strategy

PubMed, Scopus, and Web of Science were searched from inception to July 22, 2024, thereby including all available records up to the date of the search, without language or filter restrictions. Key words and Medical Subject Headings terms for “ascending aorta,” “endovascular,” and “stent-graft” were combined with Boolean operators; full strategies are provided in Table E1. Two reviewers (J.F.K. and M.G.B.) independently screened titles and abstracts, resolving discrepancies with a senior author (S.T.). In addition, experts in the field were consulted to ensure inclusion and thorough coverage of all relevant studies. In addition to the studies identified through the systematic search, we included 1 additional study based on a dataset collected and analyzed by our own research group. Although this study was unpublished at the time of the search, it has since been published and is cited accordingly.

### Inclusion and Exclusion Criteria

Inclusion criteria were all available English-language studies reporting on outcomes of aTEVAR used as treatment for diseases which originate in the ascending aorta (Ishimaru zone 0). Any type of endovascular treatment was eligible for inclusion, ie, tubular stent grafts, fenestrated stent graft, scalloped stent grafts, branched stent grafts, debranched stent grafts, and chimney placement. Studies were required to include a minimum of 5 patients.

Exclusion criteria were nonadult patients, studies with fewer than 5 patients, and diseases affecting the ascending aorta, but not originating in it (eg, retrograde extensions and diseases beyond the ascending aorta necessitating proximal landing in zone 0). If the disease origin was unclear, the study was excluded from analysis. In case of updated studies or studies describing the same study population, the most recent publication was included.

### Study Selection

Rayyan.ai was used for removal of duplicate studies and screening of abstract and title.^20^ No automation tools were used for screening of the full text. After eligibility assessment, relevant studies were stored in Mendeley reference management software to streamline the research.^21^

### Data Collection

Data extraction was performed independently by 2 authors (J.F.K. and M.G.B.), who collected relevant data from all included studies. Data were collected in Excel (Microsoft) spreadsheets. Data per study were reported under each outcome measure. In cases when studies did not report on a certain outcome measure, “?” was recorded. Discrepancies in data extraction were resolved through consensus. All study data are available from the corresponding author on reasonable request. Individual-level data will be shared in deidentified form and may require a data use agreement to protect participant privacy.

### Outcome Measures

Primary outcome measures were in-hospital, 30-day, and overall mortality. In addition, a subgroup analysis based on pathology was performed. Secondary outcome measures included complication rates, divided into intraoperative and postoperative (which includes all complications occurring after surgery and during follow-up), as well as other descriptive characteristics such as stent manufacturers, patient characteristics, procedural specifics, and hospital length of stay.

### Data Description and Analysis

Continuous data are presented using weighted mean and standard deviation (SD), median and interquartile range, weighted mean and range, or absolute values and range for complications, the latter being due to overall low occurrences. Ranges represent the number of cases reported per study. All statistical analyses were conducted using RStudio 4.3.2 (http://www.r-project.org).^22^ Weighted means and pooled SDs for continues variables (eg, age, follow-up duration, procedural time) were calculated using sample-size weighting, incorporating both within-study and between-study variation. For studies reporting medians and ranges or interquartile ranges, the sample mean, and SD were first estimated using the formulas provided by Luo and colleagues^23^ and Wan and colleagues.^24^ Proportions (eg, in-hospital mortality, 30-day mortality, and overall mortality) were pooled using weighted averages, and 95% CIs were calculated using the Clopper-Pearson Exact method.^25^ When studies did not report on specific outcome measures, analyses were performed on the data that was available. Subgroup analyses of mortality were performed per disease entity of the ascending aorta.

### Quality Assessment

Quality assessment was performed independently by 2 authors (J.F.K. and M.G.B.) The quality of the included studies and potential bias threats were evaluated using the Risk Of Bias In Non-randomised Studies of Interventions tool, assessing bias across 7 distinct domains.^26^ A GRADE certainty analysis was done for the outcome measures, to evaluate the quality of evidence and strength of its recommendations.^19^

## Results

### Study Selection

The primary literature search identified a total of 5313 studies (PubMed: 1608; Web of Science: 1249; Scopus: 2456). After removal of 2444 duplicates, 2869 studies were eligible for title and abstract screening. In total, 2655 studies were excluded on the basis of title and abstract, resulting in 214 studies eligible for full-text assessment. All 214 studies were available in full-text English.

**Figure 2:**
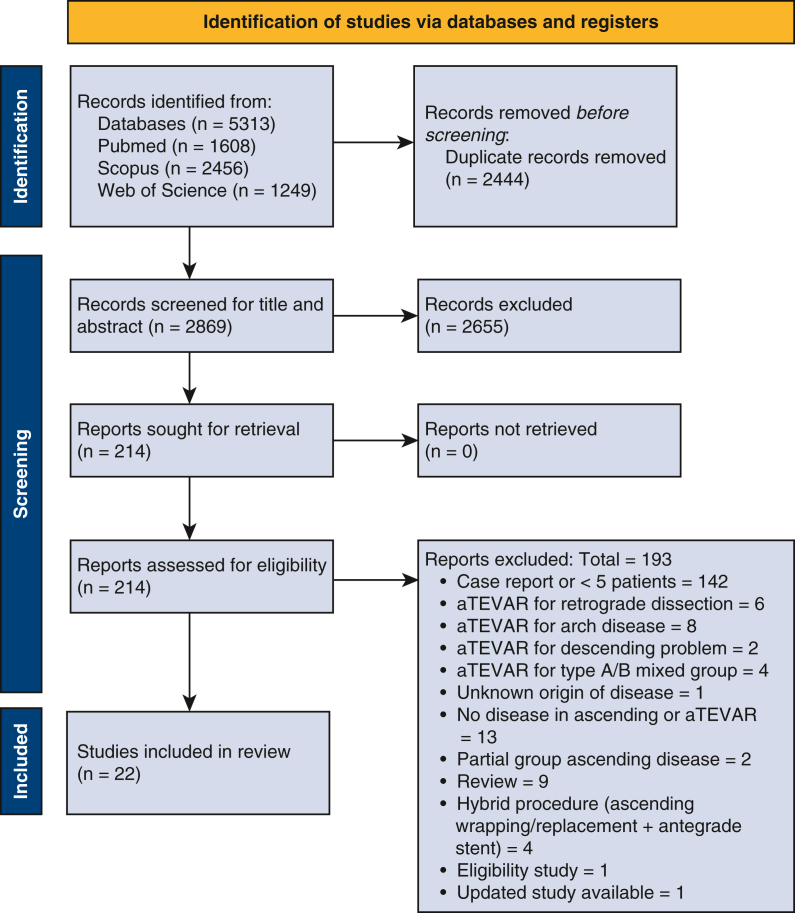
Preferred Reporting Items for Systematic Reviews and Meta-Analyses flow diagram of study selection process for endovascular treatment of diseases originating in the ascending aorta. *aTEVAR*, Ascending thoracic endovascular aortic repair.

In total, 22 studies met the inclusion criteria,^27^, ^28^, ^29^, ^30^, ^31^, ^32^, ^33^, ^34^, ^35^, ^36^, ^37^, ^38^, ^39^, ^40^^,^^E1^ including 1 study that was added postsearch, after its publication.^E2^ Figure 2 illustrates the methodologic identification for study inclusion. All included studies were single arm cohort studies. All studies analyzed data retrospectively, apart from the ARISE trial.^E3^ Table E2 depicts an overview of the study characteristics.

### Quality Assessment

All studies were determined to have an overall critical level of bias, due to the critical levels of bias in dimension 1 (bias due to confounding). All studies were scored as low in the domains 2 (bias due to selection of participants), 3 (bias in classification of interventions), 4 (bias due to deviations from intended interventions), 5 (bias due to missing data), 6 (bias in measurements of outcomes), and 7 (bias in selection of reported results).

**Figure E1:**
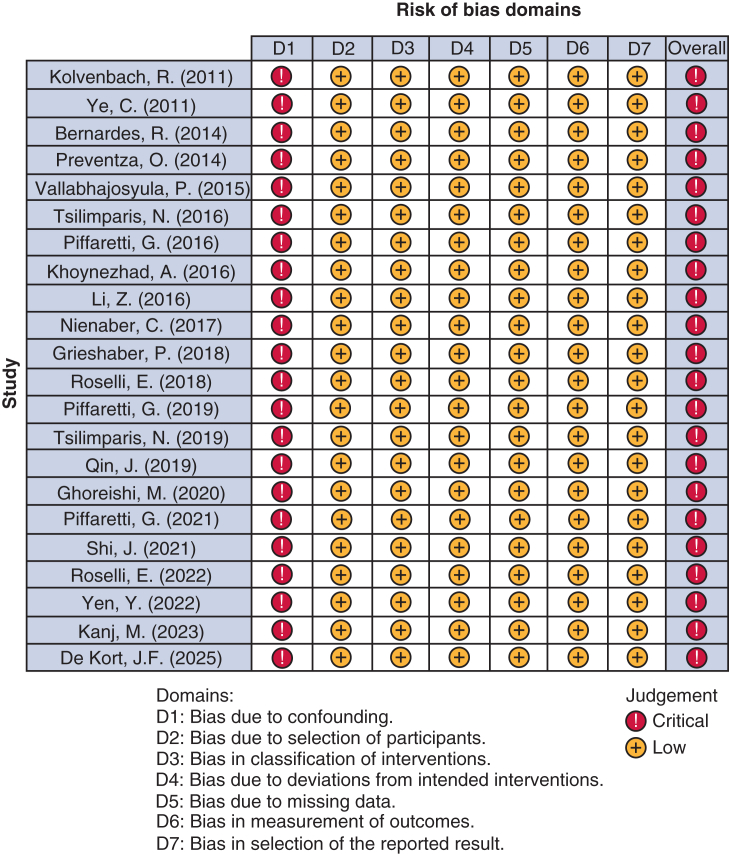
Risk Of Bias In Non-randomised Studies of Interventions tool risk of bias assessment for quality of evidence.

An overview of all bias domains and overall score for each included study is presented in Figure E1. The GRADE quality assessment with concomitant explanations for each score per domain is provided in Table E3. The GRADE assessment showed overall very low quality of evidence.

### Patient Population

A total of 356 patients were treated with aTEVAR. Of those, 211 (59.3%) patients were male and weighted mean age was 68.6 ± 10.9 years. Weighted mean follow-up time was 21.2 ± 20.3 months. Frailty scores were reported as American Society of Anesthesiologists score (3.6 ± 0.41) and EuroSCORE II (16.1 ± 7.63).^E9^^,^^E10^ Most frequently reported comorbidity was hypertension (n = 259, 72.8%), followed by coronary artery disease (n = 86, 24.2%) and diabetes (n = 86, 24.2%). Table 1 provides an overview of all the reported comorbidities and their respective prevalence rates.

**Table 1:** Baseline characteristics of patients treated with endovascular treatment of diseases originating in the ascending aorta

Patient characteristics (1):	Patients (N = 356)
Age, y, mean ± SD	68.6 (±10.9)
Follow-up (n = 293),∗ mean ± SD	21.2 (±20.3)
ASA score (n = 52), mean ± SD	3.6 (±0.41)
EuroSCORE II (n = 35), mean ± SD	16.1 (±7.63)
Male patients	211 (59.3%)
Comorbidities	
Hypertension	259
Coronary artery disease	86
Diabetes	86
Dyslipidemia	60
COPD	60
Renal insufficiency	78
Heart failure	57
Valvular disease	45
Stroke	49
Peripheral artery disease	40
Cardiac arrythmia	33
Myocardial infarction	35
Malignancy	16
Cardiac disfunction (unspecified)	11
Connective tissue disease	1
Earlier thoracic surgery	137 (38.5%)
Open aortic surgery	25 (7.0%)
Open heart surgery	27 (7.6%)
Cardiac valve replacement	15 (4.2%)
Thoracotomy (unspecified)	9 (2.5%)
TEVAR	17 (4.8%)
Transplantation	3 (0.8%)
Unspecified	46 (12.9%)
Aortic pathology	
Acute type A dissection	128 (36.0%)
Chronic type A dissection	71 (19.9%)
Pseudoaneurysm	60 (16.9%)
Penetrating aortic ulcer	41 (11.5%)
Ascending aneurysm	31 (8.7%)
Unspecified type A dissection	6 (1.7%)
Type 1a endoleak	5 (1.4%)
Intramural hematoma	3 (0.8%)
Rescue during TAVI	3 (0.8%)
Rupture	2 (0.6%)
Iatrogenic coarctation	1 (0.3%)
Anastomotic fistula†	1 (0.3%)
Unspecified	4 (1.12%)

*SD*, Standard deviation; *ASA*, American Society of Anesthesiologists; *EuroSCORE*, European System for Cardiac Operative Risk Evaluation; *TEVAR*, thoracic endovascular aortic repair; *COPD*, chronic obstructive pulmonary disease; *TAVI*, transcatheter aortic valve intervention.

∗ Patients reported on.

† After ascending replacement.

**Figure 3:**
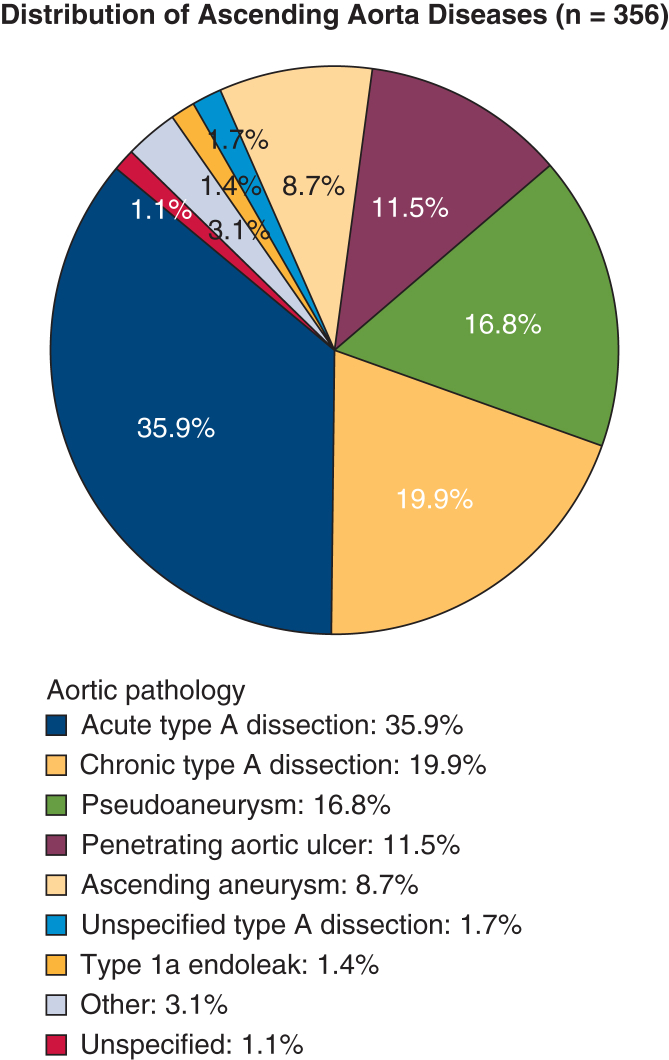
Distribution of ascending aortic diseases treated endovascularly among included studies. Other: Intramural hematoma (0.8%), rescue during TAVI (0.8%), rupture (0.6%), iatrogenic coarctation (0.3%), anastomotic fistula (0.3). *TAVI*, Transcatheter aortic valve intervention.

In total, 137 of 356 (38.5%) patients had a history of previous thoracic surgery, most commonly open aortic surgery (n = 25, 7.0%) followed by open surgery of the heart (n = 27, 7.6%). In 46 cases (12.9%), the specific type of previous surgery was not mentioned. The most frequent indication for aTEVAR was acute TAAD in 128 patients (36.0%), followed by chronic TAAD in 71 patients (19.9%) and pseudoaneurysm in 60 patients (16.9%). Table 1 depicts patient characteristics and the various ascending diseases. Figure 3 further illustrates the distribution of the various ascending aortic diseases with their respective rates.

### Procedural Specifics

The urgency of the procedure was specified in 188 procedures, and 110 (60.8%) were performed in an urgent setting. Weighted mean procedural time was 151 ± 62 minutes. Rapid ventricular pacing was the most used method to manage cardiac output (n = 119, 33.4%), followed by the use of cardiopulmonary bypass (n = 66, 18.5%). Endovascular access was primarily obtained through the femoral and iliac arteries (n = 305, 82.7%). Transapical access was used in 28 cases (7.6%). Table E4 provides an overview of the procedural specifics.

### Interventions

The most performed procedure type was tubular aTEVAR (n = 229, 64.3%) without stent graft modifications or adjunctive procedures, such as the necessity of a bypass or the placement of additional stent-grafts. Second most frequent procedure was in situ diode laser fenestration, which was performed in 58 cases (16.3%). Notably, this method of fenestration was only used in the study by Qin and colleagues.^E4^ On-table physician-modified fenestrated stent grafts were used in 20 cases (5.6%). Table 2 elaborates on the various procedure types performed throughout the studies.

**Table 2:** Types of endovascular treatment for diseases originating in the ascending aorta

Type of procedure	Number of procedures (%)
aTEVAR	229 (64.3)
Fenestrated stent graft (in situ laser diode)	58 (16.3)
SM fenestrated stent graft	20 (5.6)
SM stent graft (shortened)	11 (3.1)
Stent graft + debranching	5 (1.4)
Stent graft + chimney	4 (1.1)
Stent graft + bypass	3 (0.8)
Stent graft + chimney + bypass	3 (0.8)
Branched stent graft	3 (0.8)
Branched stent graft + bypass	3 (0.8)
Coils	3 (0.8)
Fenestrated stent graft	2 (0.6)
Occluder device	2 (0.6)
Scalloped stent graft	1 (0.3)
Scalloped and fenestrated stent graft	1 (0.3)
Fenestrated stent graft + bypass	1 (0.3)
Unspecified	7 (2.0)

*aTEVAR*, Ascending thoracic endovascular aortic repair; *SM*, surgeon modified.

### Stent Grafts

Table E5 summarizes the stent grafts used during the interventions, with a total of 293 stent grafts reported. The most frequently used stent was the GORE Conformable Thoracic Aortic Graft (n = 92, 31.4%), followed by Bolton Relay NBS thoracic stent graft (n = 63, 21.5%) and Cook Zenith Ascend thoracic endovascular graft system (n = 34, 11.6%). The manufacturer that provided the greatest number of stents was Gore Medical, with 122 (41.6%) stents. Stent diameters ranged from 30 to 50 mm and lengths ranged from 50 to 200 mm. In some cases, the manufacturer was mentioned in the study, yet the exact measurements of stents used were not specified. Another study^33^ describes the use of 14 stents but does not specify their manufacturer. Adoption of custom-made stent grafts was sparingly reported, which limits its specific analysis. All included cases had a proximal landing zone in zone 0, as the disease originated in the ascending aorta. One study involved in situ laser fenestration after extending into zone 1, targeting the brachiocephalic trunk only. No studies reported on stents with coronary fenestrations, or on endo-Bentall procedures. Most studies did not report precise landing zones or outcomes per stent graft, limiting the possibility for stratified outcome analyses.

### Primary Outcome

A total of 73 patients died across all the included studies during complete follow-up, resulting in an overall mortality rate of 20.5% (95% CI, 16.4%-25.1%). The in-hospital mortality rate across all patients was 10.7% (n = 38; 95% CI, 6.0%-12.7%), and 30-day mortality rate was 9.3% (n = 29; 95% CI, 8.4%-15.3%). A subgroup analysis of patients with TAAD (n = 183) (unspecified whether acute or chronic) showed an overall mortality of 18% (n = 33; 95% CI, 12.7%-24.3%), an in-hospital mortality rate of 9.8% (n = 18; 95% CI, 5.9%-15.0%), and a 30-day mortality rate of 10.3% (n = 19; 95% CI, 6.3%-15.7%). Among patients with pseudoaneurysms (n = 60), the overall mortality rate was 13.3% (n = 8; 95% CI, 5.9%-24.6%), with in-hospital and 30-day mortality rates of both 2.8% (n = 3; 95% CI, 1.0%-13.9%). Overall mortality rate for patients with penetrating aortic ulcer (PAU; n = 41) was 14.6% (n = 5; 95% CI, 5.6%-29.2%). In-hospital and 30-day mortality rates for PAU were 7.3% (n = 3; 95% CI, 1.5%-19.9%) and 9.8% (n = 4; 95% CI, 2.7%-23.1%). Aneurysms were seen in 16 cases, with an overall mortality rate of 25% (n = 4; 95% CI, 7.3%-52.4%), and in-hospital and 30-day mortality rates of both 12.5% (n = 2; 95% CI, 1.6%-38.4%). Mortality rates of the most prevalent indications are further detailed in Table 3.

**Table 3:** In-hospital, 30-day, and total follow-up mortality stratified by ascending aortic disease

Disease, n	In-hospital mortality (%, 95% CI)	30-day mortality (%, 95% CI)	Complete follow-up mortality (%, 95% CI)
Type A dissection (183)	18 (9.8%, 95% CI, 5.9-15.0)	19 (10.3%, 95% CI, 6.3-15.7)	33 (18.0%, 95% CI, 12.7-24.3)
Pseudoaneurysm (60)	3 (2.8%, 95% CI, 1.0-13.9)	3 (2.8%, 95% CI, 1.0-13.9)	8 (13.3%, 95% CI, 5.9-24.6)
Ascending aneurysm (16)	2 (12.5%, 95% CI, 1.6-38.4)	2 (12.5%, 95% CI, 1.6-38.4)	4 (25%, 95% CI, 7.3-52.4)
PAU (41)	3 (7%, 95% CI, 1.5-19.9)	4 (9.8%, 95% CI, 2.7-23.1)	6 (14.6%, 95% CI, 5.6-29.2)
Total (356)	38 (10.7%, 95% CI, 6.3-13.1)	29 (9.3%, 95% CI, 6.3-13.1%)	73 (20.5%, 95% CI, (16.4-25.1)

*PAU*, Penetrating aortic ulcer.

### Complications

**Figure 4:**
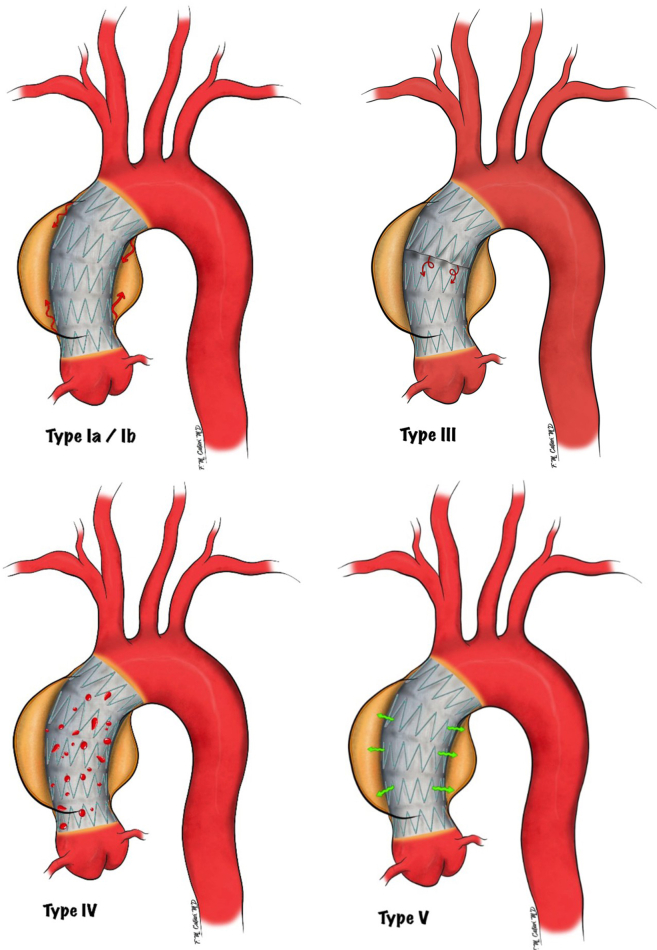
Diagram illustrating the different types of endoleak following ascending thoracic endovascular aortic repair (aTEVAR), including: Type I (proximal Ia and distal Ib attachment site leaks), Type III (graft defect or modular disconnection), Type IV (graft porosity), and Type V (endotension without visible leak).

A total of 59 intraoperative complications (16.6%, range across studies: 0-8) were documented. In total, 14 (3.9%; range, 0-3) endoleaks were observed intraoperatively, of which type Ia endoleak occurred in 8 (2.2%, range 0-2) cases; see Figure 4. Second most reported complication were conversions to open surgery, which occurred in 10 cases (2.8%; range, 0-4).

Unplanned adjunctive procedures occurred in 9 patients (2.5%), with a respective range of 0 to 3. Adjunctive procedures often consisted of unplanned transcatheter aortic valve replacement and/or the necessity for an intraoperative bypass.

A total of 148 postoperative complications (47.4%; range, 0-31) were recorded. Overall, endoleaks occurred in 37 (10.4%; range, 0-8) cases. Most common was type Ia endoleak, which occurred in 10 cases (2.8%; range, 0-2). In 20 cases (5.6%; range, 0-7) endoleak type was unclear or unspecified. Another prevalent complication was stroke (n = 27, 7.6%; range, 0-6). Late reoperations were necessary in 37 patients (10.4%; range, 0-7). Table 4 depicts the most frequently reported intra- and postoperative complications.

**Table 4:** Summary of intra- and postoperative complications after endovascular treatment of diseases originating in the ascending aorta

Complications	Total events, n	Range (min-max of events reported per study)
Intraoperative		
Endoleak	14	0-3
Ia	8	0-2
Ib	2	0-1
II	0	0
III	1	0-1
Unspecified	3	0-1
Unplanned adjunctive procedures	9	0-3
Conversion	10	0-4
Aortic insufficiency	6	0-1
Coverage of artery	5	0-3
Cerebrovascular accidents	1	0-1
Total	59	0-8
Postoperative		
Endoleak	37	0-8
Ia	10	0-2
Ib	6	0-3
II	1	0-1
III	0	0
Unspecified	20	0-7
Cerebrovascular accident	27	0-6
Respiratory failure	9	0-3
Arrythmia	9	0-4
Myocardial infarction	8	0-2
Tamponade	8	0-2
Renal failure	13	0-6
Aortic insufficiency	6	0-3
Total	154	0-31
Late reoperation	37	0-7

## Discussion

This review included 22 cohort studies (2011 onward) with 356 patients undergoing aTEVAR for disease originating in the ascending aorta (zone 0). Most patients were considered unfit for open surgery because of medical frailty, anatomic complexity (eg, multiple sternotomies), or refusal; the advanced mean age and high comorbidity burden, together with elevated American Society of Anesthesiologists and European System for Cardiac Operative Risk Evaluation II values, confirm a high surgical-risk profile. As reporting of comorbidities varied across the included series, the true burden of risk factors is likely greater. Within this context, in-hospital and 30-day mortality were 10.7% and 9.3%, respectively, and overall follow-up mortality was 20.5%, although several studies did not specify if deaths were aorta-related.^32^^,^^33^^,^^35^^,^^37^^,^^39^^,^^40^^,^E3, E4, E5, E6, E7^,^^E11^

Compared with our previous analysis confined to stent grafts strictly within the ascending aorta (in-hospital 7.3%, 30-day 7.7%, follow-up 17.0%), current mortality rates are slightly greater.^15^ This likely reflects the broader scope of the current review, which included more extensive repairs on the basis of disease origin rather than procedural definition. Publication bias present in earlier case reports may also have contributed to the more favorable estimates.

Although our previous focused systematic review helps to delineate outcomes of isolated ascending repair, the present analysis arguably reflects more closely the heterogenous real-world application of ascending endovascular repair. However, meaningful comparison between procedural strategies is limited by insufficient detail across the included studies, preventing stratification by stent type, procedural technique or extent of arch involvement. Other literature encompassing all endovascular procedures with deployment of a stent-graft in zone 0, including arch lesions, also reported lower mortality scores, with pooled 30-day/in-hospital mortality rates of 7.5%.^E12^ For specific diseases, outcomes should be interpreted with considerable caution because of small sample sizes and selected cohorts.

Among patients with TAAD, in-hospital and 30-day mortality after aTEVAR were 9.8% and 10.3%, respectively, with an overall follow-up mortality of 18%. These findings fall within the range reported in surgical cohorts (10.9%-23.6% in-hospital mortality),^12^, ^13^, ^14^ although the populations are not directly comparable: patients undergoing open repair generally have lower frailty scores and fewer prohibitive comorbidities, whereas the endovascular cohorts are more selected in anatomy and are surgically considered unfit. In this context, comparison with medically treated patients is more clinically relevant, because both groups comprise individuals for whom open surgery was not considered feasible. The distinction lies in anatomical suitability. Mortality in medically managed TAAD remains considerably greater (57.1%-60.2% in-hospital).^12^, ^13^, ^14^ Notably, the present data combine acute and chronic dissections, as most studies did not report them separately, which may affect interpretation. Follow-up mortality aligns with surgically treated cohorts (14.9%-20%) and remains below that of medically treated patients (>60%-83.9%), although these differences must be interpreted cautiously given the underlying selection biases.

For PAU, in-hospital and 30-day mortality of 7.0% and 9.8% and follow-up mortality of 14.6%, fall within ranges reported for TEVAR in other series (4.8%-14.6% early; 10.4%-22.9% follow-up).^3^^,^^E13^^,^^E14^ These mortality rates, however, include PAU diagnoses across the entire thoracic aorta.^3^^,^^E13^^,^^E14^

Similarly, observed mortality rates after endovascular repair for pseudoaneurysm (2.8% in-hospital, 13.3% follow-up) appears comparable with the 6% 30-day and 74% follow-up mortality after open repair, although comparison is limited by sparse contemporary data.^E15^

Our review only reports on 16 cases of fusiform aneurysms. Although this group is very limited and mortality rates might therefore not be representative, it shows the potential endovascular management of an ascending lesion type that is not intuitively considered as an indication for stent graft.

The postoperative type 1a endoleak rate appears to be relatively low at 2.8%. However, among reported cases, the total postoperative endoleak rate is 10.4%, with 5.6% unspecified. Given the distribution of endoleak across identified types, it is probable that a substantial proportion of these unspecified endoleaks are type 1a. A 9.0% incidence of type 1a endoleak is reported in the literature.^E12^ The results of a meta-analysis on TEVAR for various aortic arch pathologies indicate a postoperative endoleak type 1a/III rate of 8.1%.^E16^ The rates found in our review appear high; however, this could be attributed to the lack of specific stent grafts and deployment systems tailored for the ascending aorta. These results may display the off-label use of a wide variety of abdominal/conventional stent grafts in terms of sizes and the frequent need for physician modification, illustrating the need for devices specifically manufactured of ascending aorta stent-grafts. The ARISE trial, which is the first to use stent grafts specifically designed for treating TAAD, reported a notably greater endoleak rate, 26.3%, compared with the overall endoleak rate of this review. However, these are the first preliminary results, as the trial is ongoing.^E3^

The periprocedural stroke rate found in this review was 7.6%, which aligns with a reported stroke rate of 9.0% as reported in literature.^E12^ Other literature reports a periprocedural stroke (nontransient) rate after endovascular repair of the aortic arch of 4.8%, and a rate of 16.4% for TAAD managed with open surgery.^E16^^,^^E17^ Stroke is a substantial complication in ascending and arch operations for both endovascular and open surgery. Manipulation of the aortic wall with stiff guidewire and stent graft shafts, leading to the release of atherothrombotic debris, has been suggested to be a major cause of strokes.^E18^^,^^E19^

In our review, the rate of late reoperations was reported at 10.4% among cases, with 38.2% of these cases being managed endovascularly. A 30-day reoperation rate of 7.8% after open surgery for ascending aortic dissections is described in the literature.^E20^

### Limitations

Several limitations must be acknowledged. Publication bias is likely, as studies with favorable outcomes are more often submitted and accepted, especially in early single-center experiences. Patients were carefully selected on the basis of anatomy or procedural suitability despite being unfit for open repair, which may further contribute to the relatively low observed mortality.

Only single-arm cohort studies were available, precluding direct statistical comparison with open surgery or medical management, although such comparative trials are probably not ethically feasible. Variability in definitions, follow-up duration, and complication reporting introduced information bias and limits the precision of pooled estimates.

All included studies had a critical risk of bias because of unmeasured confounding, and the overall GRADE certainty of evidence was very low. Nevertheless, patient selection generally reflected real-world practice, where aTEVAR is reserved for anatomically suitable individuals at prohibitive surgical risk.

Some series lacked explicit exclusion criteria, complete follow-up, or standardized imaging schedules, possibly leading to underestimation of late complications. Sample sizes in subgroup analyses were often too small for definitive conclusions, particularly outside the TAAD group.

Finally, the procedures encompassed a wide range of devices, from simple occluders to complex branched stent grafts. This heterogeneity complicates direct comparison but mirrors current clinical practice, in which endovascular strategies are tailored to individual anatomy. As a result of limited reporting, however, stratification of outcomes by treatment type was not feasible.

## Conclusions

The published experience with aTEVAR to date shows feasibility and encouraging results, particularly in patients who are unfit for open repair because of medical frailty or previous cardiac surgery. The current literature suggests that this procedure has primarily been performed in carefully selected cases, including type A dissection or pseudoaneurysm at previous cannulation sites. However, the current knowledge is limited to a few, mainly small retrospective studies, with low level of evidence.

Although the results underscore the procedure's potential effectiveness, they also reveal a substantial rate of complications, emphasizing the need for optimized stent grafts, delivery systems, and routinization of this procedure. These complications are likely to decrease as expertise in the field continues to grow. Further prospective studies with standardized reporting and longer-term follow-up are essential to better define the role of aTEVAR in clinical practice.

## Conflict of Interest Statement

C.E.V.B. Hazenberg is or has been proctor or consultant for Gore Medical, Terumo Aortic, and Cook Medical and speaker for Artivion Inc. G. Piffaretti is a Lecturer for W.L. Gore & Associates, Terumo Aortic, and Lifetech. J. A. van Herwaarden is or has been proctor or consultant for Gore Medical, Terumo Aortic, and Cook Medical and speaker for Artivion Inc. S. Trimarchi is consultant and speaker for Medtronic Inc, W.L. Gore & Associates, and Terumo Aortic. All other authors reported no conflicts of interest.

The *Journal* policy requires editors and reviewers to disclose conflicts of interest and to decline handling or reviewing manuscripts for which they may have a conflict of interest. The editors and reviewers of this article have no conflicts of interest.

## Figures and Tables

**Table E1:** Full search string used per database and concomitant found results

Database	Search query	Results
1. PubMed	May 1, 2024 (1) July 22, 2024 (2)	1608
	(Ascend∗ OR (“zone 0”)) AND aorta) AND (“TEVAR” OR Stent OR “endoprosthesis”).	
2. Scopus	May 1, 2024 (1) July 22, 2024 (2)	2456
	(Ascend∗ OR (“zone 0”)) AND aorta) AND (“TEVAR” OR Stent OR “endoprosthesis”).	
3. Web of science	May 1, 2024 (1) July 22, 2024 (2)	1249
	(Ascend∗ OR (“zone 0”)) AND aorta) AND (“TEVAR” OR Stent OR “endoprosthesis”).	

**Table E2:** Overview of the included studies: study design, setting, and number of patients

Study	Publication y	Study type	Center/registry	Patients, n
Kolvenbach et al^27^	2011	Retrospective cohort	Single center	11
Ye et al^28^	2011	Retrospective cohort	Single center	10
Bernardes et al^29^	2014	Retrospective cohort	Single center	7
Preventza et al^30^	2014	Retrospective cohort	Single center	7
Vallabhajosyula et al^31^	2015	Retrospective cohort	Single center	5
Tsilimparis et al^32^	2016	Retrospective cohort	Multicenter	10
Piffaretti et al^33^	2016	Retrospective cohort	Multicenter	8
Khoynezhad et al^34^	2016	Prospective cohort	Single center	6
Li et al^35^	2016	Retrospective cohort	Single center	15
Nienaber et al^36^	2017	Retrospective cohort	Multicenter	12
Grieshaber et al^37^	2018	Retrospective cohort	Single center	6
Roselli et al^38^	2018	Retrospective cohort	Single center	39
Piffaretti et al^39^	2019	Retrospective cohort	GREAT registry	6
Tsilimparis et al^40^	2019	Retrospective cohort	Single center	24
Qin et al^E4^	2019	Retrospective cohort	Single center	58
Ghoreishi et al^E5^	2020	Retrospective cohort	Single center	13
Piffaretti et al^E6^	2021	Retrospective cohort	Multicenter	9
Shi et al^E7^	2021	Retrospective cohort	Single center	31
Roselli et al^E3^	2022	Prospective cohort	Multicenter	19
Yen et al^E8^	2022	Retrospective cohort	Single center	11
Kanj et al^E1^	2023	Retrospective cohort	Single center	5
De Kort et al^E2^	2025	Retrospective cohort	Multicenter	44

**Table E3:** GRADE assessment on certainty for mortality outcomes after endovascular treatment of diseases originating in the ascending aorta

Quality assessment								Patients, n	Effect			Quality	Importance
Studies, n	Design	Risk of bias∗	Inconsistency	Indirectness	Imprecision	Pubication bias	Other considerations	aTEVAR	In-hospital mortality, n (%)	30-d mortality, n (%)	Overall mortality at follow-up, n (%)		
Main outcomes													
21	Observational cohort studies	Very serious∗	Serious†	Serious‡	Serious§	Undected publication bias‖	Not applicable	356	38 (9.0%; 95% CI, 6.3-13.1)	29 (9.3%; 95% CI, 6.3-13.1)	73 (20.5%; 95% CI, (16.4-25.1)	⨁OOO Very low	Important

∗ Due to threat of potential confounders and lack of adjustment.

† Due to discrepancy in mortality ratios.

‡ Due to lack of control group and baseline risks per subgroup.

§ Due to small sample sizes.

‖ Due to discrepancies in study outcomes.

**Table E4:** Procedural characteristics: urgency, access routes, and methods of cardiac output control

Procedural characteristic	Number of patients (%)
Elective surgery	71 (39.2%)
Urgent surgery	110 (60.8%)
Procedural time, min (n = 188), mean ± SD	151 (±62)
Method of managing cardiac output	
Rapid ventricular pacing	119 (33.4%)
Mechanical induction	57 (16.0%)
Cardiopulmonary bypass	66 (18.5%)
Adenosine	2 (0.6%)
Unspecified	112 (31.5%)
Type of access	
Femoral/iliac artery	305 (82.7%)
Transapical	28 (7.6%)
Axillary artery	24 (6.5%)
Subclavian	7 (1.9%)
Carotid	5 (1.4%)

*SD*, Standard deviation.

**Table E5:** Stent graft devices used to treat diseases of the ascending aorta by manufacturer and model

Manufacturer/device	Number of stent grafts used (%)
GORE TAG	92 (31.4)
Bolton NBS	63 (21.5)
Cook Medical Zenith Ascend	34 (11.6)
Cook Medical Zenith TX2	30 (10.2)
GORE EXCLUDER	15 (5.1)
GORE ASG	15 (5.1)
Hercules	15 (5.1)
Medtronic Valiant PS-IDE	7 (2.4)
Medtronic Valiant	5 (1.7)
Medtronic Talent	1 (0.3)
CookZenith EVAR iliac limb	1 (0.3)
Optimed	1 (0.3)
Aegis	unknown amount
Ancurell	unknown amount
CookProForm	unknown amount
Unspecified	14 (4.8)
